# Aberrant expression of redox protein Ape1 in colon cancer stem cells

**DOI:** 10.3892/ol.2014.1864

**Published:** 2014-02-10

**Authors:** DEBAO LOU, LINA ZHU, HUAWEI DING, HAI-YAN DAI, GANG-MING ZOU

**Affiliations:** 1 Department of Pharmacy, Shanghai Eighth People’s Hospital, Shanghai 200235, P.R. China; 2 Department of Ophthalmology, Renji Hospital, School of Medicine, Shanghai Jiao Tong University, Shanghai 200127, P.R. China; 3 Shanghai Cancer Institute, Shanghai Jiao Tong University, Shanghai 200240, P.R. China; 4 Shanghai Institute for Pediatrics Research, Xin Hua Hospital, Shanghai Jiao Tong University Shanghai of Medicine, Shanghai 200092, P.R. China

**Keywords:** colon cancer, E3330, Ref-1, 5-fluorouracil, MDR1, ABC-G2

## Abstract

Ape1 is an important redox protein, essential for specific cytokine-induced signal transduction. Ape1 signaling is also important in regulating the growth of cancer cells, including colon cancer cells. The present study investigated whether Ape1 signaling plays a role in the regulation of colon cancer stem cell (CCSC) growth. The results showed that Ape1 was aberrantly expressed in CCSCs, as determined by quantitative (q)PCR assay. A laser confocal microscopy assay demonstrated that the Ape1 protein was mainly distributed in the nuclei, but not the cytoplasm, of the CSCs. Treatment of CCSCs with Ape1 redox inhibitor (E3330) significantly affected growth *in vitro*. In colon cancer xenograft mice, *in vivo* administration of E3330 enhanced tumor responses to the chemotherapeutic drug, 5-fluorouracil (5-FU). Furthermore, the combination of E3330 and 5-FU evidently increased the cytotoxicity of 5-FU in CSC growth. In the qPCR assay, the CCSCs were demonstrated to express the dominant ATP-binding cassette sub-family G member 2 (ABC-G2), but not the multidrug resistance 1, genes. Thus, we hypothesized that drug resistance in CCSCs is mediated by ABC-G2. Since CSCs are involved in cancer metastasis, the Ape1 inhibitor may be a potential agent in the inhibition of colon cancer growth and metastasis.

## Introduction

Ape1, also named Ref-1, is a multifunctional protein involved in apurinic/apyrimidinic (AP) endonuclease DNA base excision repair (BER) and redox activity. Ape1 is essential for specific cytokine-induced signal transduction, including that of interleukin-21 (1) and cluster of differentiation (CD)40L (2). CD40L triggers its receptor, CD40, which consequently mediates Ape1 nuclear translocation in human Burkitt’s lymphoma cells (2). Ape1 is ubiquitously expressed at high basal levels. Previous studies in which Ape1 was disrupted using a gene targeting strategy have been used to determine its function *in vivo* (3). Embryos without Ape1 expression die *in utero* between implantation and day 6.5, indicating normal embryonic development requires Ape1 (4). This gene is aberrant in various cancer cells (1). Our previous study demonstrated the aberrant expression of Ape1 in pancreatic cancer (5) and its role in tumor angiogenesis (6)

Redox balance underlies cellular homeostasis. Cancer initiation and progression have been associated with the disruption of redox balance and oxidative stress (7). Previously, Funato *et al* (8) demonstrated that reactive oxygen species can modulate signaling through the Wnt/β-catenin pathway. The study highlighted new insights into the cross-talk between redox and Wnt/β-catenin signaling in normal physiology and cancer. The Wnt signaling pathway may be regulated by redox signaling through redox-sensitive association of nucleoredoxin with dishevelled. A number of solid tumors contain substantial fractions of hypoxic cells that are relatively resistant to radiation therapy and certain cytotoxic drugs. Ape1, a nuclear protein, maintains the reduced state of Fos and Jun and promotes binding to activator protein 1 (AP-1). Nuclear extracts of HT29 colon cancer cells exposed to hypoxia show a markedly increased Ape1 protein content. Elevation of Ape1 mRNA levels occur as an early event following the induction of hypoxia and persists when cells are restored to a normally oxygenated environment (9). In addition, Ape1 Asp148Glu polymorphisms may be associated with the increasing risk of colorectal cancer (CRC) in a Turkish population (10).

Colon cancer is the second leading cause of cancer mortality in the United States, and 50% of patients with colon cancer develop synchronous or metachronous liver metastasis (11). Bleomycin (BLM) is used to treat various types of cancer, which generate cytotoxic double-strand breaks and abasic AP sites in DNA. The human Ape1 acts on abasic or 3′-blocking DNA lesions generated by ionizing radiation (IR) or BLM. It has been shown that cells are markedly sensitized to BLM cytotoxicity by partial Ape1 deficiency (30% of normal levels), while resistance is largely restored by the expression of the unrelated yeast AP endonuclease, Apn1. It has also been demonstrated that apoptosis induced by BLM under Ape1 deficiency is partially p53-dependent in HCT116 colon cancer cells. Thus, the suppression or inhibition of Ape1 may be more efficacious as an adjuvant for BLM than for IR cancer therapy, particularly for tumors with a functional p53 pathway (12). These results pose the question of whether Ape1 expression is also important in the drug resistance of cancer stem cells (CSCs) in CRC. In the present study, Ape1 expression was examined in colon CSCs (CCSCs). It was found that CCSCs express dominant levels of the Ape1 gene and that the Ape1 inhibitor, E3330, enhances the chemotoxicity of 5-fluorouracil (5-FU) to these cells.

## Materials and methods

### Cell culture

The human colon cancer HT29 cell line was purchased from the American Type Culture Collection (Manassas, VA, USA). The cells were cultured in Dulbecco’s modified Eagle’s medium (DMEM) supplemented with 10% fetal bovine serum, 100 U/ml penicillin and 100 U/ml streptomycin (Invitrogen Life Technologies, Carlsbad, CA, USA).

### Tumor sample collection

Fresh specimens of CRC tissue and adjacent non-cancerous tissues were obtained from patients who underwent surgical resection of preliminary CRCs and/or liver metastasis at the Department of Surgery, Shanghai Eighth People’s Hospital (Shanghai, China). The study was approved by the Institutional Review Board of the hospital. A histological assessment was performed by pathologists using the World Health Organization standard grading system, with four categories (well-, moderately-, and poorly-differentiated and undifferentiated), was used (13).

### Isolation of CCSCs

Fresh colorectal tumor specimens were immediately minced on ice, suspended in DMEM/F12 medium and dissociated with collagenase (both Invitrogen Life Technologies) and hyaluronidase (Calbiochem, La Jolla, CA, USA). Enzymatically disaggregated suspensions were filtered and washed three times with PBS and red blood cells were removed by Histopaque-1077 (Sigma-Aldrich, St. Louis, MO, USA). The resulting single tumor cells were placed under stem cell conditions in serum-free DMEM/F12 supplemented with human recombinant EGF and basic FGF (Invitrogen Life Technologies), and cultured on Ultra Low Attachment plates (Corning Inc., Corning, NY, USA). Single-cell suspensions in stem cell medium (1:10,000 cells/well) were analyzed for tumor sphere formation and numbers in each well were quantified after 14 days. Next, tumor spheres were disaggregated and reseeded to evaluate self-renewal by formation of secondary tumor spheres. Isolation of CD133^+^/ESA^+^ CCSCs was performed by magnetic-activated cell sorting (MACS) or fluorescence-activated cell sorting (FACS).

### Quantitative (q)PCR

Total RNA was extracted from the samples using TRIzol according to the manufacturer’s instructions (Invitrogen Life Technologies). RNA was solubilized in ddH_2_O, and the nucleic acid concentration was measured by a Nano Drop 2000 (Thermo Fisher Scientific, Waltham, MA, USA). cDNA was prepared using the PrimeScript kit (Takara Biotechnology (Dalian) Co., Ltd., Dalian, China). In each case, random and oligo(dT) primers were used. qPCR analysis was performed with an ABI 7300 (Applied Biosystems, Inc., Foster City, CA, USA), using SYBR Premix EX Taq as the reaction reagent. The relative quantities of the genes were calculated with β-actin as a reference, using the following formula: $\text{Relative quantity of gene}=2^{\left[-(\text{CtGene}-\text{Ct}\beta -\text{actin})\right]}$. Primer sequences are listed in Table I.

### Laser confocal microscopy

For experiments on Ape1-Flag localization, a sequential protocol of immunofluorescence was used, based on labeled goat anti-mouse isotype-specific secondary antibodies. The cells were fixed in 4% (wt/vol) paraformaldehyde for 20 min at room temperature, permeabilized for 5 min with PBS-0.25% (wt/vol) Triton X-100 and incubated for 30 min with 5% normal goat serum in PBS-0.1% (wt/vol) Triton X-100 (blocking solution) to block the non-specific binding of the antibodies. The cells were then incubated with the mouse monoclonal anti-FLAG (IgG1; 1:1,000; Sigma-Aldrich) or -APE1 (IgG2b; 1:30; Cell Signalling Technology, Danvers, MA, USA) antibodies, in blocking solution, for 2.5 h. Following washing, the cells were incubated for 90 min with Alexa Fluor 488-conjugated goat anti-mouse, IgG1 or Alexa Fluor 546-conjugated goat anti-mouse, IgG2b (1:200; Invitrogen Life Technologies) secondary antibodies. The preparations were then washed with PBS three times for 5 min each in the dark. Nuclei were stained by 5 min of incubation in a 300-nM solution of 4′,6-diamidino-2-phenylindole dihydrochloride (Sigma-Aldrich) in PBS. Next, the preparations were washed three times in PBS for 5 min. The microscope slides were mounted onto slides in Mowiol 4-88 (Sigma-Aldrich), supplemented with DABCO (1:5; Sigma-Aldrich), as an antifade reagent. Coverslips were visualized by a Leica TCS SP laser scanning confocal microscope (Leica, Mannheim, Germany).

### Cell growth assay of CCSC in vitro in response to 5-FU and E3330

CCSCs were cultivated with a cell density of 1×10^4^ cells per well in a 96-cell plate overnight. On the second day, the cells were treated with E3330 alone (concentration of 1 μM) or with the addition of various doses of 5-FU (1 or 5 μg/ml) for 48 h. Next, MTT assays were performed to analyze cell viability.

### Animal and tumor models

Female, four-week-old (mu^+^/nu^+^) mice were obtained from the Animal Center of Chinese Academy of Science (CAS, Shanghai, China) and maintained under the Institutional guideline of CAS. The CSCs that formed spheres were dissociated by collagenase and washed with PBS twice. The dissociated sphere cells (5×10^5^) were injected subcutaneously with Matrigel (BD Biosciences, Franklin Lakes, NJ, USA) in a total volume of 100 μl. Tumor sizes were calculated once a week for 20 weeks according to the following formula: Tumor size=π/6×larger diameter×smaller diameter. Chemotherapy sensitization was assessed, and well-established tumors were generated by subcutaneous injection of sphere cells. Six mice per group were treated intraperitoneally with 5-FU (15 mg/kg/day, 5 days a week for 2 weeks) alone or in combination with intratumoral injections of E3330 (1.5 mg/kg/day, 5 days a week for 2 weeks).

### Statistical analysis

Statistical analysis was performed using the SPSS 9.0 statistical software package (SPSS, Inc., Chicago, IL, USA), and Student’s t-test was used to test the probability of significant differences between samples. P<0.05 was considered to indicate a statistically significant difference.

## Results

### Ape1 is expressed in CCSCs

**Figure 1:**
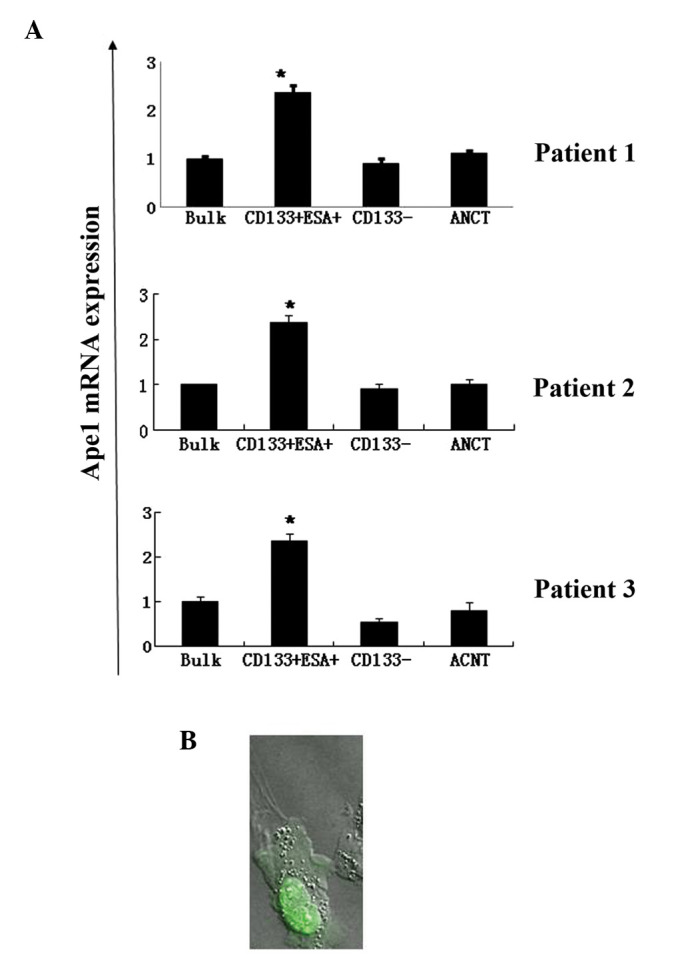
Expression of Ape1 gene in CD133^+^/ESA^+^ CCSCs examined by quantitative (q)PCR and laser confocal microscopy. (A) CD133^+^/ESA^+^ CCSCs were isolated from fresh colon cancer tissue by FACS. The total RNA was extracted and qPCR was performed. (B) Ape1 gene expression (green) in CCSCs examined by laser confocal microscopy. ^*^P<0.05, vs. control cells. CCSCs, colon cancer stem cells; FACS, fluorescence-activated cell sorting.

To elucidate the role of Ape1 redox signaling in CSC regulation, the expression level of Ape1 was first examined in CCSCs. CD133^+^/ESA^+^ CCSCs were isolated from bulk cancer cells by FACS or MACS. The expression of the Ape1 gene in the CCSCs was examined by qPCR assay in the cells isolated from three independent patients. As shown in Fig. 1, all cells were found to express higher levels of the Ape1 gene compared with CD133^−^/ESA^+^ non-stem cells or adjacent non-cancerous tissues (Fig. 1A). Ape1 protein expression was further examined in these CSCs using laser confocal microscopy. The expression of Ape1 protein in the CCSCs was confirmed by laser confocal microscopy, and was mainly distributed within the nuclei of the CCSCs (Fig. 1B).

### Inhibition of Ape1 redox function by E3330 affects CCSC growth

**Figure 2:**
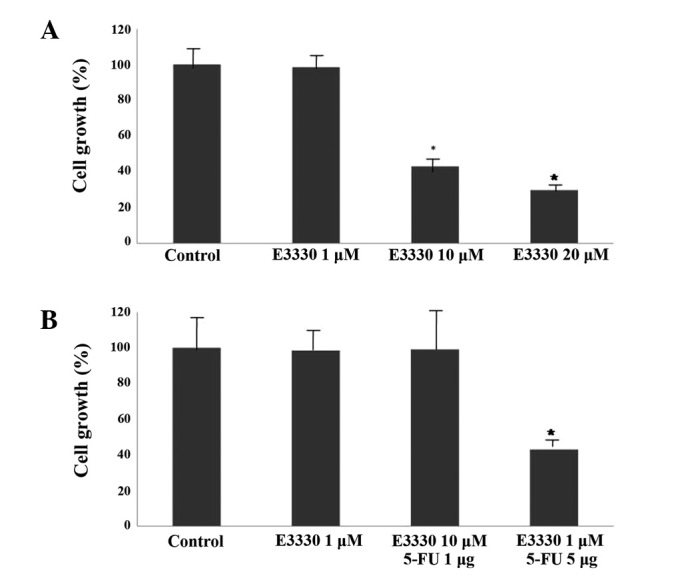
Effect of Ape1 redox inhibitor on CD133^+^/ESA^+^ CCSC growth. (A) Results showing dose-dependent inhibition of CCSC growth by E3330. CD133^+^/ESA^+^ CCSCs were treated with various doses of E3330 for 72 h. MTT assays were then performed to analyze cell viability. (B) E3330 enhanced the cytotoxic effect of 5-FU in suppressing CCSC growth *in vitro*. E3330 at a non-cytotoxic dose (1 μM) was applied in this experiment. Data are presented as the mean ± SD of three independent experiments. ^*^P<0.05 vs. control. CCSCs, colon cancer stem cell; 5-FU, 5-fluorouracil.

E3330 is a specific inhibitor of the Ape1 redox domain (3). Our previous study described the role of E3330 in inhibiting the growth of digestive tract cancer cells, such as pancreatic cancer cells (4). In the present study, the role of E3330 in the regulation of CSC growth was examined. The treatment of CCSCs with E3330 was found to significantly reduce the growth of cells in cultures treated with an E3330 concentration of 10 μM (Fig. 2A). Moreover, the addition of a non-cytotoxic (i.e. cell growth-inhibiting) dose of E3330 (1 μM) was found to significantly enhance the chemotoxicity of 5-FU in CCSCs *in vitro* (Fig. 2B).

### Combination of E3330 with 5-FU enhances the cytotoxicity of 5-FU in colon cancer chemotherapy

**Figure 3:**
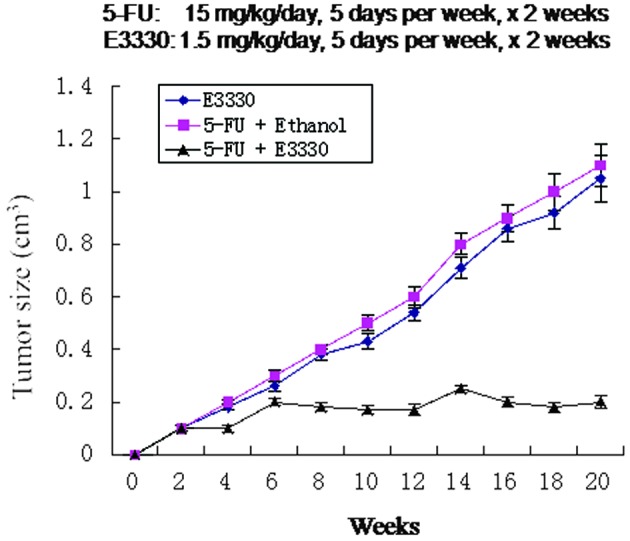
Combinated effect of E3330 and 5-FU in the suppression of CD133^+^/ESA^+^ CCSC growth *in vivo*. Tumor size following intratumoral injection of ethanol alone (vehicle control), 5-FU or 5-FU/E3330 post-treatment. Data are presented as mean tumor size ± SD of four independent sets of five tumors per group. Each set of tumors was exhibited using cells from different tumors. 5-FU was administered at 15 mg/kg/day, 5 days per week for 2 weeks; and E3330 administered at 1.5 mg/kg/day, 5 days per week for 2 weeks. 5-FU, 5-fluorouracil; CCSC, colon cancer stem cell.

To test the hypothesis that the combination of E3330 with 5-FU enhances the cytotoxicity of 5-FU in colon cancer chemotherapy *in vivo*, E3330 was analyzed to investigate whether it enhances the chemotherapuetic efficiency in CCSC xenograft models. 5-FU is a standard drug in the chemotherapy of colon cancer. Tumor xenografts generated by sphere cell injection were exposed to intraperitoneal administration of 5-FU plus intratumoral injections of ethanol or E3330. The tumor response to chemotherapeutic drugs was enhanced by E3330, whereas ethanol, as a control pretreatment, did not prevent tumor outgrowth. Specifically, the combined administration of E3330 and 5-FU resulted in a marked antitumor effect, whereas 5-FU alone did not (Fig. 3).

### Multidrug resistance 1 (MDR1) and ATP-binding cassette sub-family G member 2 (ABC-G2) gene expression in CD133^+^/ESA^+^ CCSCs

**Figure 4:**
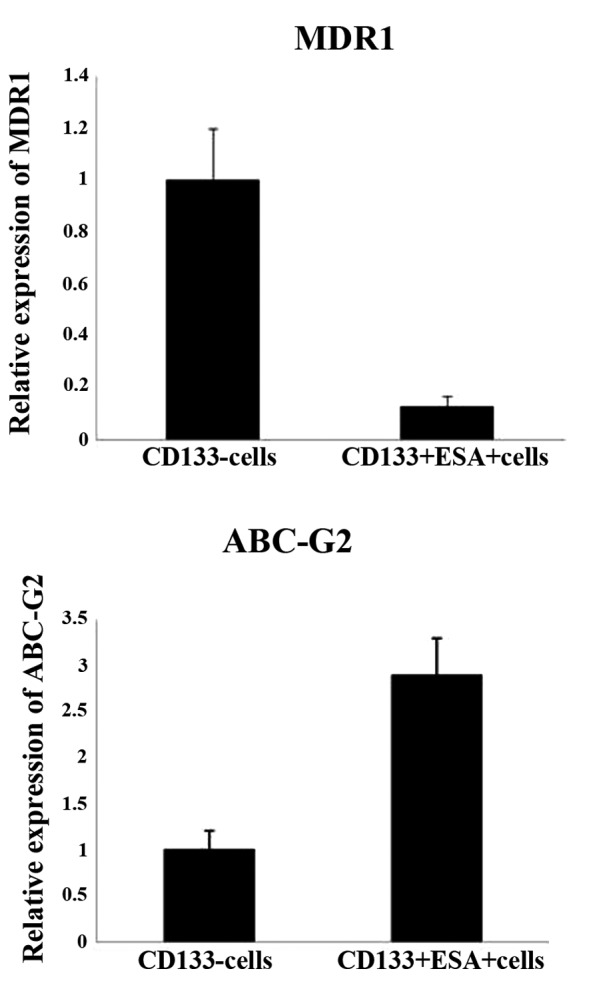
Drug resistance gene expression in CCSCs. MDR1 and ABC-G2 gene expression was examined by quantitative (q)PCR in CD133^+^/ESA^+^ CCSCs isolated from fresh CRC tissue. Data are presented as the mean ± SD of values for three patients. ^*^P<0.05, vs. CD133^−^ cells. CCSC, colon cancer stem cell; CRC, colorectal cancer; MDR1, multidrug resistance 1; ABC-G2, ATP-binding cassette sub-family G member 2.

The overexpression of human Ape1, a key enzyme in the DNA BER pathway, is often associated with tumor cell resistance to various anticancer drugs. It has been previously indicated that Ape1 stably interacts with the Y-box-binding protein 1 (YB-1) and acts as its coactivator for the expression of the multidrug resistance gene, MDR1, thereby causing drug resistance. Ape1 is also stably associated with the basic transcription factor, RNA polymerase II, and the coactivator, p300, on the endogenous MDR1 promoter (11). MDR1 gene expression was examined by qPCR in CD133^+^/ESA^+^ CCSCs. Compared with CD133^−^/ESA^+^ colon cancer cells of tumor tissue, CD133^+^/ESA^+^ CCSCs were found to express extremely low levels of the MDR1 gene. Therefore, other drug resistance genes were examined and the ABC-G2 gene was found to be expressed dominantly in CD133^+^/ESA^+^ CCSCs, but not in CD133^−^/ESA^+^ cancer cells derived from cancer tissue (Fig. 4).

## Discussion

Ape1 is a multifunctional enzyme involved in DNA BER and protein redox regulation. This redox protein is overexpressed in various types of tumor tissues and cells, including lung (15) and bladder (16) cancer cells. However, the mechanism of expression of this redox protein in CSCs currently remains unclear. In the present study, the aberrant expression of Ape1 was identified in CCSCs by the examination of primary human colon cancer tissues. Previously, CSCs have been shown to be associated with drug resistance and the failure of chemotherapy in cancer, but the mechanism in this regard has not been fully demonstrated (17). Overexpression of the Ape1 gene in pathological stem cells is associated with drug resistance. It has been previously demonstrated that drug-induced Ape1 acetylation, which is mediated by p300, enhances the formation of the acetylated Ape1/YB-1/p300 complex on the MDR1 promoter. Enhanced recruitment of this complex increases MDR1 promoter-dependent luciferase activity and its endogenous expression in HEK 293T cells (14). However, the present study showed that Ape1 may not regulate drug resistance through MDR1, but alternatively through ABC-G2 in CSCs.

The ABC-G2 gene is also highly expressed in hematopoietic stem cells, but this gene is turned off in the majority of committed progenitor and mature blood cells (18). CSCs possess high levels of ABC transporters. MDR1, which is also named ABC-B1, is a transmembrane transporter system, which actively pumps cytotoxic drugs out of the cell. Although MDR1 acquired *in vitro* is different from MDR1 acquired *in vivo*, it has important consequences on the metastatic behavior and cellular phenotype. The MDR1-negative human colonic cancer HT29 cell line is more malignant than its MDR1-overexpressing variant (HT29 MDR1-positive). When implanted subcutaneously into SCID mice, undifferentiated signet ring carcinomas are produced by HT29 MDR1-negative cells, while tumors with tubular structures, but without signet ring cells, are formed by HT29 MDR1-positive cells (MDR1 overexpression in HT29 colon cancer cells grown in SCID mice) (19). The results of the current study are consistent with this hypothesis, and CCSCs were found to express extremely low levels of the MDR1 gene, but high levels of ABC-G2. Thus, we hypothesized that ABC-G2, but not MDR1, is key in the drug resistance of CSCs.

5-FU is one of the major drugs in chemotherapy for CRC, but CCSCs are resistant to 5-FU (20). CCSCs may be expanded as tumor spheres *in vitro* using a serum-free medium containing EGF and bFGF. Such tumor spheres contain CSCs, cancer progenitors and early precursors, presenting the best-characterized method to expand an enriched population of tumorigenic cells (21). Wnt activity is high in CD133-positive DLD1 colon cancer cells compared with CD133-negative DLD1 colon cancer cells. The Wnt activity of CD133-positive colon cancer cells may be upregulated by 5-FU, while blocking this activity may reverse the drug sensitivity of these cells to 5-FU (22). Therefore, the combination of Ape1 and Wnt inhibitors exhibits an improved effect in inhibiting colon cancer cell proliferation. This indicates that the combination of these inhibitors may improve the efficacy of future CRC therapy. We aim to analyze the association between Ape1 and Wnt signaling in CCSCs in future studies.

In summary, the present study identified the aberrant expression of Ape1 in CCSCs, and the inhibition of its redox activity is likely to enhance the cytotoxicity of 5-FU against these cells.

## Figures and Tables

**Table I:** Primer sequences for qPCR.

Gene name	qPCR primers
Ape1	
Forward	ACT TCA GGA GCT GCC TGG ACT
Reverse	AAT GCA GGT AAC AGA GAG TGG GA

qPCR, quantitative PCR.
